# The Need for Integrating Addiction Medicine and Hepatology

**DOI:** 10.1001/jamanetworkopen.2022.13022

**Published:** 2022-05-02

**Authors:** Lorenzo Leggio, M. Katherine Jung

**Affiliations:** National Institute on Drug Abuse, National Institutes of Health, Baltimore, Maryland; National Institute on Alcohol Abuse and Alcoholism, National Institutes of Health, Bethesda, Maryland; National Institute on Alcohol Abuse and Alcoholism, National Institutes of Health, Bethesda, Maryland

Vannier and colleagues^1^ reported the results of a large retrospective cohort study that aimed to investigate the incidence of alcohol-associated liver disease (ALD) development in patients with alcohol use disorder (AUD) who received a medication for AUD. Using electronic medical records, these authors identified 9635 patients with AUD, among whom 3906 patients were treated with pharmacotherapy for AUD. Specifically, Vannier and colleagues^1^ selected patients who had received 1 of the 3 medications approved by the US Food and Drug Administration (FDA) for AUD (acamprosate, disulfiram, and naltrexone). Furthermore, they included patients who had received topiramate, gabapentin, or baclofen, which are medications that have not been approved by the FDA for AUD but may be used as off-label drugs given the clinical evidence supporting their efficacy.^2–4^ The authors found that patients who received pharmacotherapy for AUD were less likely to have a diagnosis of ALD and to develop hepatic decompensation. Among patients who already had a diagnosis of cirrhosis, those who received pharmacotherapy for AUD were less likely to experience hepatic decompensation. Furthermore, in patients who experienced a hepatic decompensating event despite having received pharmacotherapy for AUD, the time to decompensation after cirrhosis diagnosis was significantly longer compared with patients who did not receive pharmacotherapy.^1^

In patients with AUD and ALD, alcohol is the etiological factor associated with the development of ALD; hence, treating AUD may prevent the development of ALD and may be associated with improved liver-related outcomes in those with ALD, as Vannier and colleagues^1^ reported. The take-home message from this study may seem obvious, but its clinical and public health importance cannot be overemphasized. One of the most challenging aspects in treating patients with AUD is that many patients do not receive evidence-based treatments despite the availability of such treatments. This lack of treatment is alarming and is a reason that the results of this study are important, now more than ever. Alcohol use continues to increase globally, and the rates of AUD, including the most severe form of unhealthy alcohol use, have also increased. Recent data indicate that the number of deaths involving alcohol increased between 2019 and 2020 (the first year of the COVID-19 pandemic) from 78 927 to 99 017 (relative change, 25.5%); the age-adjusted rate also increased from 27.3 to 34.4 per 100 000 (relative change, 25.9%).^5^

Excessive alcohol use is a leading cause of mortality and morbidity.^6^ Among the medical consequences of unhealthy alcohol use, a cardinal one is ALD. Parallel to the increase in alcohol use and AUD, the rates of ALD continue to increase, especially among young people and women.^2^ The number of deaths with an underlying cause of ALD increased between 2019 and 2020, from 24 106 to 29 504 (relative change, 22.4%).^5^

The treatment of AUD includes psychosocial and/or pharmacological interventions. Acamprosate, disulfiram, and naltrexone (oral and intramuscular), are approved by the FDA for AUD treatment, and a meta-analysis has found support for the effectiveness of naltrexone and acamprosate in patients with AUD.^7^ Furthermore, recent guidelines from the American Association for the Study of Liver Diseases highlight and recommend the use of acamprosate in patients with AUD and ALD.^2^ This endorsement is consistent with the lack of reported cases of hepatotoxicity in patients who were treated with acamprosate and with the pharmacokinetic profile of this drug given that acamprosate is not metabolized by the liver and is excreted unchanged by the kidneys.

Furthermore, some medications that have been approved by the FDA for other indications have shown potential effectiveness in the treatment of AUD; although they are not approved for AUD, these drugs are sometimes used off-label. The most promising of these off-label medications are topiramate and gabapentin, which are recommended as potential second-line treatments for AUD by the American Psychiatric Association.^3^ Furthermore, some clinical studies have shown promising findings for baclofen, especially in patients with AUD and ALD, and the recent American Association for the Study of Liver Diseases guidelines recommend the potential off-label use of baclofen in patients with AUD and ALD, at least in those without hepatic encephalopathy.^2^

Although effective pharmacotherapy medications for AUD are available, including those that are FDA-approved, they are not widely used in clinical practice. Results from the 2019 National Survey on Drug Use and Health indicated that, among 14.1 million adults with AUD in the previous year, only 7.3% reported receiving any treatment for AUD and only 1.6% reported receiving evidence-based medications for AUD.^8^ This scenario is even more alarming in patients with AUD and comorbid ALD, a population with clinically serious medical consequences from chronic, excessive alcohol use. Treating AUD, the underlying cause of liver disease, should be the logical priority and yet, even in this particular patient population, the use of a medication to treat AUD seems to be more of an exception than the rule. For example, a recent retrospective cohort study using data from the US Department of Veterans Affairs showed that, among patients with AUD and cirrhosis, only a small number received behavioral (12%), pharmacological (0.4%), or both (1%) treatments for AUD; those who received behavioral and/or pharmacological treatment presented with a substantial reduction in incidence of hepatic decompensation and all causes of mortality.^9^

The study by Vannier and colleagues^1^ is in line with these previous studies.^9^ The findings suggest that, in patients with AUD and ALD, there are substantial beneficial outcomes to treating AUD, such as preventing the development of ALD and, among those who already have ALD, improving liver-related outcomes.^1^

A key reason for the problem of lack of treatment, despite its availability, is that there is a paucity of well-developed, multidisciplinary care models that integrate hepatology and addiction medicine. Such integration is critical for the proper treatment and care of patients with AUD and ALD. There are several barriers that prevent integrative care from happening systematically, as reviewed extensively elsewhere.^10^ First, many patients do not see AUD as a medical disorder; in those who do, the stigma and shame of having AUD often prevent them from seeking treatment. Second, clinicians often do not receive enough training in addiction to allow them to comfortably manage these patients or to at least identify the problem and ask for a consult. Third, there are various challenges at the system and administration levels, including organizational and logistical barriers, limited resources allocated to mental health and addiction care, health insurance coverage inadequacies, and inequalities in health care.^10^

The number of patients with AUD who received pharmacotherapy in the study by Vannier and colleagues^1^ was higher than in previous studies.^8,9^ This outcome is promising and was attributed by the authors to the availability of dedicated addiction services within their hospital system (Mass General Brigham).^1^ Improved treatment access is certainly evidence that, with the right organizational structure, a wider use of pharmacotherapy for AUD is possible. But sadly, the promising clinical setting in which Vannier and colleagues^1^ performed their retrospective cohort study does not reflect that of many other clinical settings.^10^

There is a need for a fundamental paradigm shift in treating patients with AUD and in developing multidisciplinary integrated models of treatment for those with AUD and ALD. This shift will require unified and synergistic efforts across several disciplines, from hepatology and addiction medicine to health care management and science policy, to have a similar favorable impact as the present study.
